# Marked mucosal lipid shifts in treatment refractory inflammatory bowel disease: a lipidomic study

**DOI:** 10.1186/s12876-025-03944-6

**Published:** 2025-05-20

**Authors:** Øystein K. Moe, Qian Gao, Dawei Geng, Einar Jensen, Rasmus Goll, Oddmund Nestegard, Mona D. Gundersen, Jon R. Florholmen, T. Moritz

**Affiliations:** 1 Research Group of Gastroenterology and Nutrition, Department of Clinical Medicine, UiT The Arctic University of Norway, Tromsø, Norway; 2 Department of Gastroenterology, Division of Internal Medicine, University Hospital of North Norway, Tromsø, Norway; 3 Department of Internal Medicine, Hammerfest Hospital, Hammerfest, Norway; 4 Novo Nordisk Foundation Center for Basic Metabolic Research, Faculty of Health and Medical Sciences, University of Copenhagen, Copenhagen, Denmark; 5 Natural Products and Medicinal Chemistry Research Group, Department of Pharmacy Faculty of Health Sciences, UiT The Arctic University of Norway, Tromsø, Norway; 6 Department of Internal Medicine, Vestre Viken Hospital, Hønefoss, Norway; 7 Department of Geriatric Medicine, Division of Internal Medicine, University Hospital of North Norway, Tromsø, Norway; 8 Swedish Metabolomics Centre, Department of Forest Genetics and Plant Physiology, Swedish University of Agricultural Sciences, Umeå, Sweden

**Keywords:** Biological therapy, Inflammatory bowel disease, Non-response, Lipids

## Abstract

**Background:**

Mechanisms causing non-response to biological agents in IBD remain to be fully understood. Thus, we aimed to characterize the lipid profile in treatment refractory non-immunogenic patients with adequate trough-levels.

**Methods:**

Patients with IBD refractory to treatment with anti-tumour necrosis factor or vedolizumab were included from a Norwegian translation study. Mucosal lipid profiles were compared to reference groups. The reference groups included treatment naïve IBD patients with moderate to severe disease at debut who later achieved remission or response on antiTNFs, IBD patients treated to remission with biological agents, and healthy normal controls. Lipidomics analyses were performed on mucosal biopsies. Statistical analyses of lipid levels were carried out using generalized least squares. Lipidomics data were log2-transformed and auto-scaled before analysis. *P*-values were adjusted using Benjamini- Hochberg procedure to control the false discovery rate (FDR).

**Results:**

Proinflammatory lipids including ceramides and sphingomyelins and protective lipids like glycerophosphocholines and glycerophosphoethanolamines were significantly decreased in treatment refractory UC patients compared to treatment naïve UC patients with moderate to severe disease. Compared to controls, major changes in ceramides (Cer), hexosyl ceramides (HexCer), sphingomyelins (SM), glycerophosphocholines (PC), glycerophosphoethanolamines (PE) and glycerophosphoserines (PS) were observed in treatment refractory UC patients. Refractory CD patients showed minor changes compared to the other CD-groups. There were no significant differences in the mucosal lipid levels of IBD patients in remission compared to normal controls.

**Conclusions:**

The mucosal lipid profile of treatment refractory UC shows marked shifts compared to treatment naïve UC at debut with moderate to severe inflammation. These are novel findings which possibly indicate dynamic processes of long-standing mucosal inflammation. The mucosal lipid profile of IBD patients in remission seems to be similar to the normal state.

**Supplementary Information:**

The online version contains supplementary material available at 10.1186/s12876-025-03944-6.

## Background

The pathophysiological events in inflammatory bowel disease (IBD) are possibly consequences of a dysregulated intestinal immune response with activation of the inflammasome [1]. The molecular understanding of the inflammasome in IBD has increased rapidly during the last two decades mostly due to novel -omics technology enabling description of the complex systems involved in immunology [1, 2]. In recent years the role of lipids in IBD has received attention. Manfredy et al. reported increased levels of eicosanoids in ulcerative colitis (UC) [3], whereas Diab et al. studied the metabolic profiles of pro-and anti-inflammatory mediators in various inflammatory states of UC [4–6]. Later, several reports have been published on lipidomics of both main forms of IBD [2, 7].

One of the most challenging issues in IBD is the understanding of pharmacodynamic mechanisms in patients who do not respond to biological therapy [8]. We have recently published a study on IBD patients in separate disease states and the mucosal transcripts of interleukin (IL) 17, IL23 and tumour necrosis factor (TNF) [9]. There were no observed differences between treatment refractory and treatment naïve IBD patients. To further assess the refractory state a lipidomic approach seemed warranted.

The lipidome consists of a large number of different lipid classes, where many have putative roles as pro- or anti-inflammatory mediators, and thereby are of interest in IBD. Sphingolipids and glycerophospholipids are of particular interest as previous studies have shown significant alterations of these lipids in IBD [5, 7].

Ceramides (Cers) constitute a family of sphingolipids with more than 50 specific molecular species possessing different functions [10]. Their properties have been regarded as mainly proinflammatory, but some Cers seem to exhibit both pro -and anti-inflammatory functions [11]. Experimental murine and in vitro cell studies as well as studies on colonic mucosa from UC patients have shown higher levels of Cers in active colitis compared to normal mucosa [5, 12].

Sphingolipid signalling is also considered as an important mediator and regulator of inflammation and TNF-related signalling [13]. Cers, sphingomyelins (SM) and their downstream products such as sphingosine-1-phosphate are widely involved in IBD related signalling pathways such as toll-like receptor (TLR) pathways and the Janus kinase/signal transducer and activator of transcription (JAK/STAT) pathway [14]. Moreover, sphingosine-1-phosphate receptor inhibitors are promising small molecule drugs recently approved by FDA for treatment of UC [15].

This study aims to elucidate the lipid profile and pathways involved in the treatment refractory state. To our knowledge this is the first report of the mucosal lipid profile in IBD patients refractory to biological therapy.

## Methods

### Patients and biopsy sampling

Patients aged ≥ 18 years were recruited in the time period 2014–2021 from 3 clinical centers in Norway (Gastrointestinal units at the hospitals of Hammerfest, Ringerike (Hønefoss) and University Hospital North Norway in Tromsø) as a part of an ongoing prospective study—Advanced Study of Inflammatory Bowel disease (ASIB- study). Most of the presently reported patients were part of an earlier publication [9], but missing biopsies for lipidomics analysis resulted in a reduced number in the present study.

All participants gave informed and written consent in accordance with the Helsinki declaration. Approval including the use of biobank was granted by the Regional Committee of Medical Ethics of Northern Norway Ref no: 1349/2012.

### Baseline characteristics

#### Diagnosis and clinical grading of activity

The diagnosis of UC and CD was based on established clinical, endoscopic, radiological and histological criteria [16]. The activity and extent of disease was evaluated using the clinical scoring systems ulcerative colitis activity index (UCDAI) [17], Crohn’s Disease Activity Index (CDAI) [18] and the Montreal classification of IBD [19]. Endoscopic findings in CD was assessed by the clinician and categorized as remission, mild, moderate or severe activity.

#### Criteria for refractory IBD to biological therapy

As described in our previous publication [9], treatment refractory IBD include pharmacodynamic non-response (primary and secondary) to biological agents and all of the following criteria: adequate treatment duration, therapeutic levels of biologics in the serum, no pathogenic bacteria in stool samples and disease activity assessed both endoscopically (inflammation and/or ulcer) and clinically (UCDAI-score > 2 or CDAI -score > 150).

Minimum adequate treatment duration for anti-TNFs varied between a minimum of 8 weeks (infliximab, IFX), 12 weeks (adalimumab, ADA) and 14 weeks (golimumab, GOL). Treatment with vedoluzimab (VDZ) for at least 14 weeks was required for inclusion [9].

Primary non-response is defined as lack of effect from the onset of treatment, while secondary non-response is loss of response during treatment [20].

### Reference groups

Treatment naïve IBD patients with moderate to severe disease at debut, IBD patients treated to remission by biologic agents and normal (healthy) controls constituted the reference groups.

Samples for reference were collected in the following settings:

- Treatment naïve IBD patients at debut of disease with endoscopically moderate to severe disease [21, 22]. Only samples from patients who within the first year after debut were in need of biologic therapy and achieved response or remission were included.
- IBD patients on biologic treatment who were in remission at the time of sampling.
- Normal (healthy) controls at cancer screening.

UC remission was defined as treatment to remission by biologics with Mayo endoscopic score 0–1, UCDAI ≤ 1 and normalized mucosal TNF transcript [23, 24]. Treatment response in UC included a UCDAI improvement of at least 3 [25]. For CD, treatment to remission included CDAI < 150, endoscopic remission and normalized mucosal TNF transcript [26]. Response in CD was defined as a CDAI score improvement of at least 70 [18]. Normal controls were subjects performing a cancer screening examination with no clinical, endoscopic or histological signs of intestinal disease.

### Tissue samples

Mucosal biopsies were sampled with standard forceps from either the region with the most severe inflammation (refractory and treatment naïve patients) or from normal mucosa (normal controls and patients in remission). For patients in remission the samples were taken from the area with previously most severe inflammation.

The biopsies dedicated to analysis by liquid chromatography and mass spectrometry (LC–MS) were immediately dry frozen in a cryotube at –70 °C. The dry weight of the biopsies ranged from 2 to 8 mg. All biopsies were at first kept at –70 °C in Tromsø until shipment by air courier to Copenhagen in a sealed styrofoam container containing dry ice.

### Lipidomics

Sample extraction was done using a modified Folch method previously published to extract lipids [27]. In brief, intestinal biopsies were extracted by adding the following solutions: 0.9% NaCl salt, 10 mg/L stable isotope internal standard solution (IS) EquiSPLASH (Avanti Polar Lipids, Inc, Alabama, USA) and chloroform: methanol mixture (1:1, v:v) prior to 10 s of vortex. Samples were then homogenized with ceramic beads 2.8 mm at 30 Hz for 3 min by a TissueLyser II from QIAGEN (Hilden, Germany) and transferred to Eppendorf tubes for 30 min of incubation on ice. Samples were spun in a centrifuge at 14,000 rpm, for 3 min at 4 °C. The lower phase was collected to LC vial with inserts for lipidomics analysis and stored in −80°C freezer prior to LC–MS analysis.

The samples were analyzed with an ultra-high-performance liquid chromatography quadrupole time-of-flight mass spectrometer (UHPLC-QTOFMS). The UHPLC system was a 1290 II Infinity system (Agilent Technologies, Waldbronn, Germany). Chromatographic separations were performed on an Acquity UPLC® BEH C_18_ column (2.1 mm × 100 mm, 1.7 µm) (Waters, Milford, MA, USA). The mobile phase A consisted of H_2_O + 1% NH_4_Ac + 0.1% HCOOH and mobile phase B of ACN:IPA (1:1, v/v) + 1% NH_4_Ac + 0.1% HCOOH. (B) were used as the mobile phases for the gradient elution. The linear gradient was as follows: from 0 to 2 min 35–80% B, from 2 to 7 min 80–100% B and from 7 to 14 min 100% B. Each run was followed by a 7 min re-equilibration period under initial conditions (35% B). The flow rate was 0.4 ml min^−1^. The column was heated to 50 °C, and the injection volumes were 1 μL. The UHPLC was coupled to a Bruker timsTOF Pro mass spectrometer (Bruker Daltonics, Bremen, Germany). All analyses were performed in both positive ion mode and negative ion mode using a VIP-HESI ion source. The scan range was 50–1000 m/z at a scan speed of 2 Hz. Quality control was performed throughout the dataset by including blanks, pooled samples, extracted standard samples and control serum samples.

The lipidomics LC–MS data files were pre-processed with Metaboscape v. 2021b (Bruker Daltonics, Bremen, Germany) and LipidBlast (version 68, http://prime.psc.riken.jp/compms/msdial/main.html#MSP) and normalized with internal standards. Lipids with > 30% missing values across all samples and relative standard deviation > 30% in pooled samples have been excluded. Missing values were imputed with 0.2 of lowest detected value for each lipid.

### Lipid profiles

The changes in lipid profiles were further evaluated by the tool BioPAN (https://lipidmaps.org/biopan/). With BioPAN it is possible to explore which lipid metabolic pathways cause the differences in lipid profiles.

### Statistical analysis

For baseline characteristics, differences among groups were tested using ANOVA for normal distributed variables, Kruskal–Wallis test for non-normal distributed variables, and Fisher’s exact or Chi-Squared test for categorical variables.Comparisons of lipid levels among groups were carried out using generalized least squares. Lipidomics data were log2-transformed and auto-scaled before analysis. *P*-values were adjusted using Benjamini- Hochberg procedure to control the false discovery rate (FDR). Uniform Manifold Approximation and Projection (UMAP) was applied to visualize the clusters in data.

A two-tailed *p*-value below 0.05 was considered statistically significant. All statistical analysis was performed in R-4.1.2.

## Results

The study included 37 UC patients, 35 CD patients and 12 normal controls. There were 21 refractory UC patients (7 female/14 male, average age 40,2 years, range 18–71 years) and 14 refractory CD patients (4 female/10 male, average age 41,5 years, range 18–56 years).

Of the 35 treatment refractory IBD patients, 22 were primary non-responders and 13 were secondary non-responders. They were either treated with antiTNFs (33 patients) or vedoluzimab (2 CD patients).

Five reference groups were included from the ASIB biobank (supplementary figure S1).

1) Eight untreated UC patients at debut with moderate to severe disease who achieved remission/response on antiTNFs; 2) Nine untreated CD patients (like group 1 but with CD); 3) Eight UC patients in remission; 4) Twelve CD patients in remission; and 5) Twelve normal controls. Characteristics are described in Table 1.

**Table 1:** Characteristics of UC and CD patients with non-response to biological treatment, treatment naïve, remission, and normal controls

Patient groups	UC non-response	CD non-response	UC treatment naïve	CD treatment naïve	UC remission	CD remission	Normal controls	*p* value
Number	*n* = 21	*n* = 14	*n* = 8	*n* = 9	*n* = 8	*n* = 12	*n* = 12	
Age	40.2 (18–71)	41.5 (18–56)	27.1 (22–39)	37.4 (22–58)	44.6 (20–76)	35.3 (22–52)	54.9 (19–83)	0.002
Sex (female/male)	7/14	4/10	4/4	5/4	1/7	5/7	5/7	0.577
Smoking current/earlier/never	2/6/7	2/7/3	0/1/5	1/2/4	1/4/2	1/1/5	2/6/3	0.542
Area involved (UC) E1/E2/E3^a^	1/10/10		1/4/3		0/3/5			0.781
Area involved (CD) L1/L2/L3/L1 + L4^a^		1/4/9/0		2/2/4/1		3/5/4/0		0.447
Duration of disease (years)	8.2 (1—35)	12.2 (3–40)			9.4 (2–28)	8.6 (2–24)		0.473
Treatment duration (weeks)	98.7 (17–270)	165.1 (21–634)			157 (73–266)	131.5 (50–321)		0.159
Non-response to anti-TNF/VDZ	21/0	12/2						0.153
Primary/secondary	12/9	10/4						0.617
Mucosal TNF	17,467 (5300–61,100)	19,836 (7600–46,400)	17,500 (12,600–27,500)	30,444 (4000–63,400)	^b^3769 (350–6500)	^b^3467 (200–6100)	5608 (300–11,400)	< 0.001
UCDAI/CDAI score	9.1 (3–12, *n* = 19)	263 (155–483, *n* = 12)	9.6 (6–12)	220 (176–250, *n* = 3)	0.4 (0–1, *n* = 7)	45 (2—114, *n* = 6)		< 0.001
MAYO score (UC) Endoscopic activity (CD) remission/mild/moderate/severe	2.6 (2–3)	0/6/4/4	2.5 (2–3)	0/0/2/0 (*n* = 2)	0,4 (0–1, *n* = 7)	12/0/0/0		< 0.001
Calprotectin mg/kg	985 (140–3000)	871 (60–3000)	893 (240–1460, *n* = 5)	562 (25–1520)	43 (20–80, *n* = 6)	41 (20–185, *n* = 10)	< 25 (*n* = 1)	< 0.001
Fecal bacterial culture performed (yes/no)	11/10	5/9						0.533
Concentration of biologic agent measured (yes/no)	19/2	12/2						1.000

Values are in actual number or mean (range). For further details, see text. ^a^The Montreal classification for IBD. ^b^Mucosal TNF expression below 7500 copies/µg total RNA was a selection criterion for these groups

### General overview of the lipidomics analysis

**Fig. 1:**
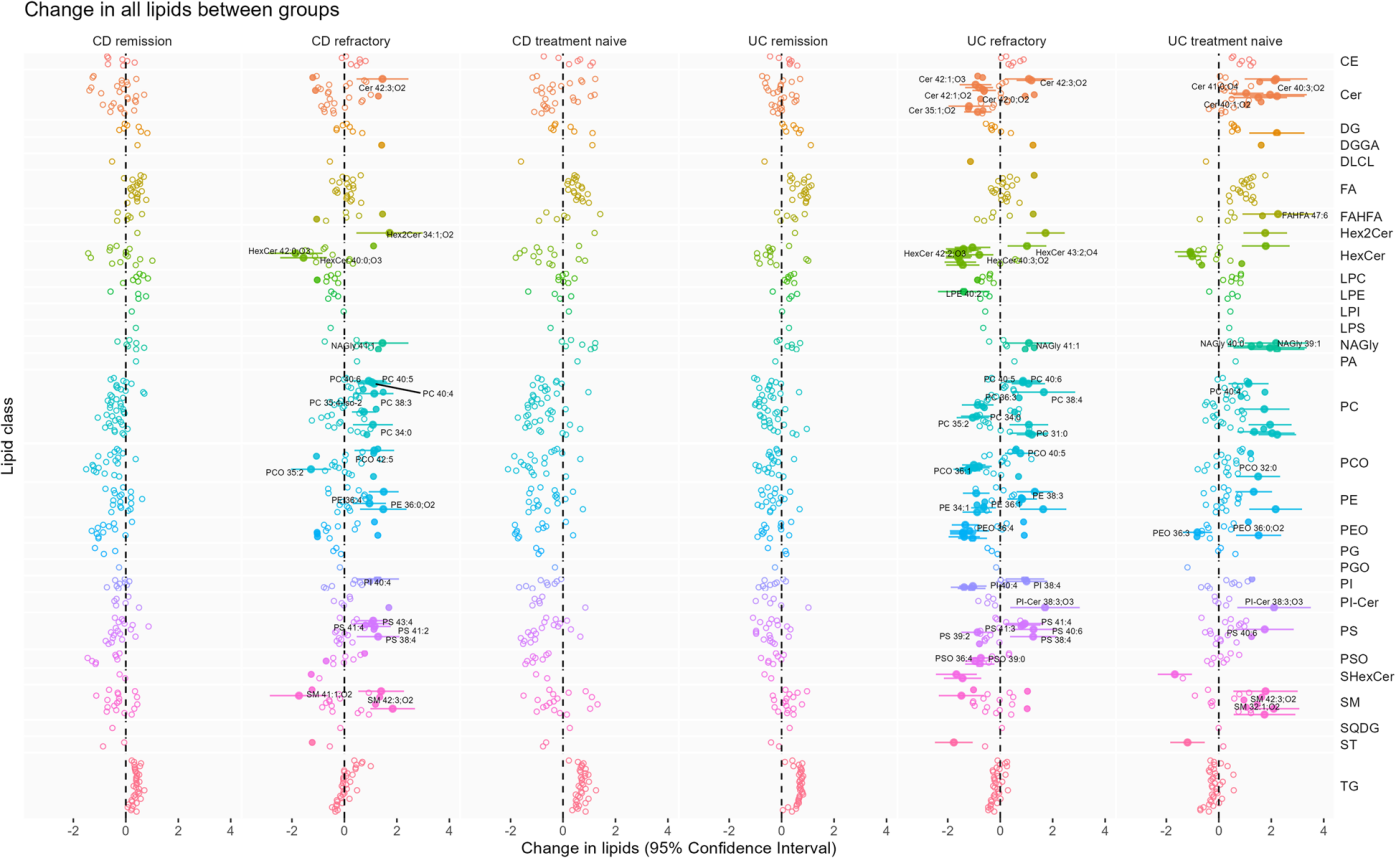
Change in lipids in CD and UC patient groups compared to normal controls. The change is calculated from generalized least squares coefficients and Benjamini–Hochberg procedure was used for multiple testing correction. Significance:empty circle, non-significant; filled circle, *p* < 0.05; filled circle with CI, *p* < 0.01

With the lipidomics analysis in total 584 different lipids were annotated, 367 in positive ion mode, 217 in negative ion mode. After removing lipids with relative standard deviation > 30% in pooled samples, 302 lipids were further evaluated for assessment of differences in mucosal lipid profiles between the different patient classes. UMAP was applied to explore the potential clusters in data (supplementary figure S2). Normal controls, CD remission and UC remission group were quite similar and seemingly clustered together. The other groups were relatively heterogeneous, especially the CD refractory and CD treatment naïve group. Furthermore, specific changes in lipids in CD and UC patient groups compared to normal controls are shown in Fig. 1.

### Mucosal lipid profiles in ulcerative colitis

**Fig. 2:**
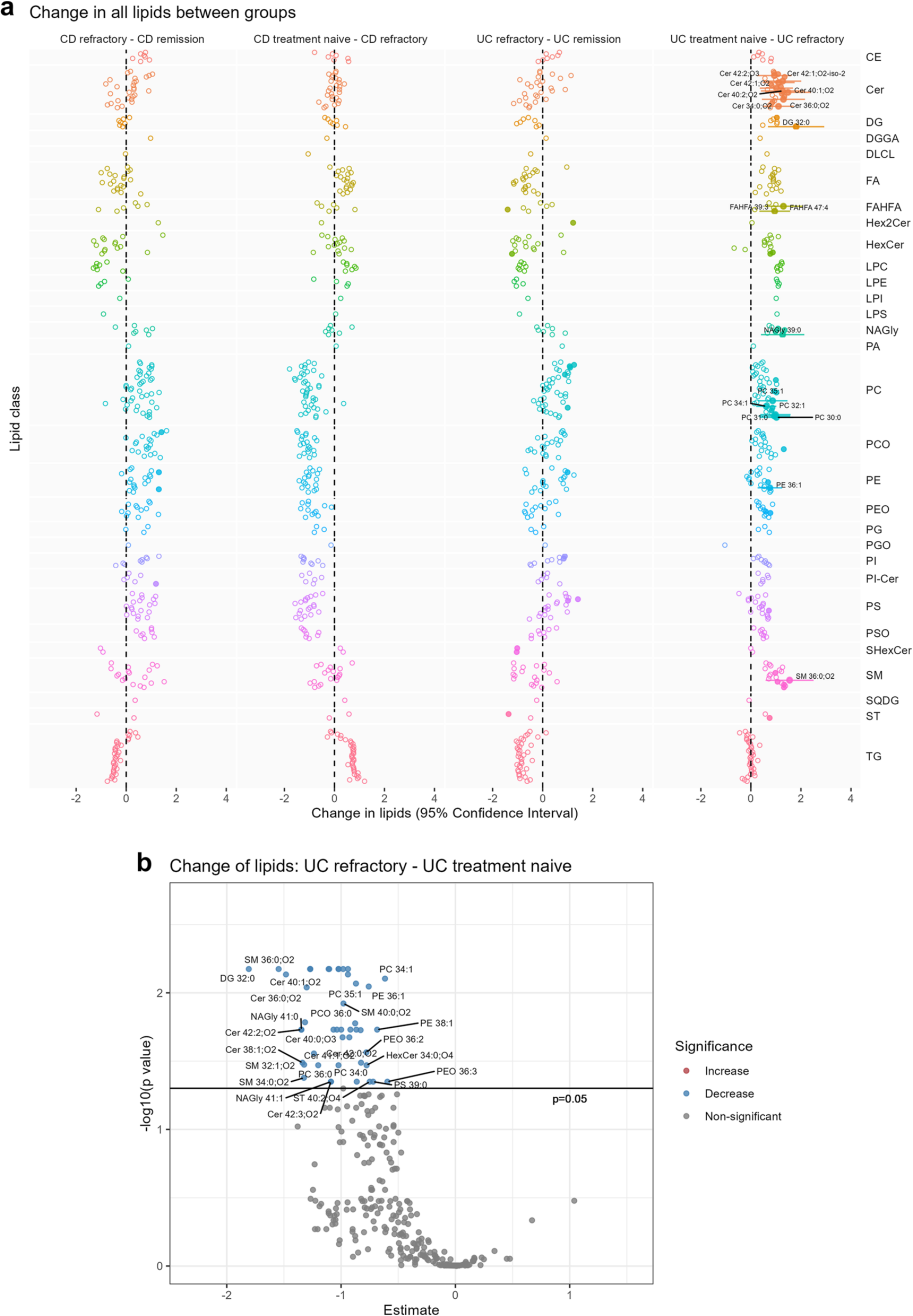
**a** Comparisons of lipid profiles among CD and UC patient groups and (**b**) volcano plot of change in lipids in UC refractory compared to UC treatment naïve. The change is calculated from generalized least squares coefficients and Benjamini–Hochberg procedure was used for multiple testing correction. Significance: empty circle, non-significant; filled circle, *p* < 0.05; filled circle with CI, *p* < 0.01. **a** Change in all lipids between groups. **b** Change of lipids: UC refractory – UC

UC refractory patients showed major changes in ceramides (Cer), hexosylceramides (HexCer), sphingomyelins (SM), glycerophosphocholines (PC), glycerophosphoethanolamines (PE) and glycerophosphoserines (PS) compared to normal controls (Fig. 1, supplementary figure S3a). Treatment naïve UC patients exhibited elevated Cer and SM levels compared to controls (Fig. 1, supplementary figure S3b). Decreased levels of Cers, SMs, PCs and PEs were detected in UC refractory patients compared to treatment naïve UC patients (Fig. 2a and b). UC patients in remission presented no significant differences to normal controls (Fig. 1).

The lipid networks generated using BioPAN were performed for UC in remission, refractory and treatment naïve group compared to normal controls. Compared to normal controls, the different groups of UC demonstrated diversity in activated and suppressed lipid pathways (supplementary figure S4). The refractory group and patients in remission showed activated conversion of PE- > PS and PC- > DG, respectively. The refractory and treatment naïve groups both showed suppressed reaction PC- > PS- > PE, which could explain the changed ratios between PC and PE. Other suppressed reactions in refractory patients were PS- > PE- > PC- > DG and PE- > PC- > PS. Compared to normal controls, PC:PE ratio significantly decreased in refractory patients (Z = −1.857). Although not significant, the same trend of decrease was shown in treatment naïve patients (Z = −1.484) (supplementary figures S4 and S5). Predicted genes encoding the enzymes responsible for the lipid pathways are shown in Table 2.

**Table 2:** Genes predicted to be involved in the activation or suppression of lipid pathways in UC groups compared to normal controls found using BioPAN

Group	Type	Reaction chains	Z-score	Predicted genes
Remission	Active	PC- > DG	1.787	
	Suppressed	DG- > PC	1.78	*CHPT1*
Refractory	Active	PE- > PS	3.149	*PTDSS2*
	Suppressed	PC- > PS- > PE	3.89	*PTDSS1, PISD*
		PS- > PE- > PC- > DG	3.013	*PISD, PEMT*
		PE- > PC- > PS	2.932	*PEMT, PTDSS1*
Treatment naïve	Active			
	Suppressed	PC- > PS- > PE	2.265	*PTDSS1, PISD*

Fatty acid network analysis for UC refractory, treatment naïve and remission compared to normal controls show both similarities and differences between the three UC groups (supplementary figure S6). The predicted genes involved in fatty acid pathways in UC refractory compared to normal controls are depicted in Table 3.

**Table 3:** Genes predicted to be involved in the activation or suppression of fatty acid pathways in UC refractory compared to normal controls found using BioPAN

Type	Reaction chains	Z-score	Predicted genes
Active	FA(18:1)- > FA(20:1)	4.961	*ELOVL3*
	FA(18:2)- > FA(20:2)- > FA(20:3)- > FA(20:4)- > FA(22:4)	3.434	*ELOVL5, FADS1, FADS1, ELOVL5, ELOVL2*
	FA(20:4)- > FA(22:4)	3.231	*ELOVL5, ELOVL2*
Suppressed	FA(20:2)- > FA(20:3)	2.263	*FADS1*
	FA(18:0)- > FA(18:1)- > FA(18:2)- > FA(18:3)	2.237	*SCD1, FADS2, FADS2*
	FA(18:1)- > FA(18:2)	2.096	*FADS2*
	FA(16:1)- > FA(18:1)- > FA(18:2)- > FA(18:3)- > FA(20:3)	1.804	*ELOVL5, ELOVL6, FADS2, FADS2, ELOVL5, ELOVL7*
	FA(18:0)- > FA(20:0)	1.793	*ELOVL1, ELOVL7, ELOVL3*

Prediction of genes suggests that *ELOVL5* and *SCD* were shifted in all three groups while *FADS2* only changed in UC refractory and treatment naïve group. Interestingly, *ELOVL3*, which is responsible for maintaining lipid homeostasis [28], was predicted to change in both refractory and treatment naïve group.

### Mucosal lipid profile in Crohn’s disease

CD refractory patients showed major changes in PEs, SMs, PSs, HexCers, SMs and PCs compared to normal controls (Fig. 1, supplementary figure S7a). The two CD refractory patients treated with vedoluzimab had previously failed on antiTNFs and were not outliers. Excluding them from the dataset showed no significant changes. There were no significant large scale changes between treatment naïve CD patients and normal controls (Fig. 1, supplementary figure S7b), nor between refractory and treatment naïve patients (Fig. 2, supplementary figure S7c). There were no significant differences between CD patients in remission and normal controls (Fig. 1).

Detailed lipid pathway study between CD refractory and controls shows active conversion of PE- > PS and suppressed conversion of PE- > PC- > PS (Z- score 3.461), PC- > PS- > PE, PS- > PE- > PC- > DG and PE- > PC- > DG (supplementary figure S8). Thus, the PC:PE ratio in CD refractory was significantly reduced compared to normal controls (supplementary figure S5).The predicted genes involved in fatty acid pathways in CD refractory compared to normal controls are shown in Table 4.

**Table 4:** Genes predicted to be involved in the activation or suppression of lipid pathways in CD refractory compared to normal controls found using BioPAN

Type	Reaction chains	Z-score	Predicted genes
Active	PE- > PS	1.832	*PTDSS2*
Suppressed	PE- > PC- > PS	3.461	*PEMT, PTDSS1*
	PC- > PS- > PE	3.065	*PTDSS1, PISD*
	PS- > PE- > PC- > DG	2.841	*PISD, PEMT*
	PE- > PC- > DG	2.186	*PEMT*

## Discussion

In this study we found a significant difference in the levels of key lipids between treatment naïve moderate to severe inflammation and a treatment refractory state in ulcerative colitis. To the best of our knowledge, these are novel findings which possibly indicate a change in immunological mechanisms during prolonged inflammation and treatment for IBD. There was no overlap between these two groups as all patients with newly diagnosed moderate to severe inflammation either responded or achieved remission on subsequent treatment with antiTNFs. Furthermore, there were no significant changes in the levels of lipids between IBD patients in remission and normal controls. Thus, the lipid profile of a mucosa in remission seems to approximate the normal state.

The majority of Cers (Cer 36:0;O2, Cer 38:1;O2, Cer 40:0;O3,Cer 40:1;O2, Cer 42:0;O2, Cer 42:2;O2 and Cer 42:3,O2) and some SMs (SM 32:1;O2, SM 34:0;O2, SM 36:0;O2 and SM 40:0;O2) were decreased in the mucosa of refractory UC patients compared to the treatment naïve UC patients (see Fig. 2b). These sphingolipids are associated with pro-inflammatory pathways in IBD [29]. Diab et. al demonstrated increasing levels of Cer(d18:1/24:0) and Cer(d18:1/24:2) in a stepwise manner from remission to active inflammation in ulcerative colitis [5]. Cer(d18:1/24:0) could represent Cer 42:1;O2 which was significantly decreased in UC refractory compared to normal controls (see supplementary figure S3a). Also, Cer(d18:1/24:2) may represent Cer 42:3;O2 which was decreased in UC refractory compared to treatment naïve UC patients (see Fig. 2b).

The relative depletion of Cers and SMs in the refractory group suggests a change in inflammatory mechanism.

Additionally, we detected reduced levels of PCs (PC 34:0, PC 34:1) and PEs (PE 36:1, PE 38:1) in the UC refractory group compared to treatment naïve UC patients (see Fig. 2b). A previous study have shown higher levels of PE 38:1 in active UC compared to the quiescent state [5]. Thus, this may also be indicative of altered mechanisms in the treatment refractory mucosa. The PC:PE ratio compared to controls was also decreased in the refractory UC patients (see supplementary figure S5). PCs and PEs are the most abundant phospholipids in mammalian cell membranes and the mucus layer of the gut, thus ensuring a protective mucosal barrier [14, 30]. Furthermore, lower PC:PE ratio has been reported to be associated with the induction of the unfolded protein response and cell death [31], and therefore a potential cause of the structure and function change in IECs in IBD patients. Taken together, these findings are suggestive of a weakened barrier affecting both the mucus layer and intestinal epithelial cells (IEC) in patients refractory to biological therapy.

With lipid network analysis we also detected suppressed reactions leading to a decreased PC:PE ratio compared to controls in the UC refractory patients. The expression of *PEMT* was predicted to be decreased and *PTDSS2* to be increased in UC refractory patients (see Table 2). Similar changes of expression have been reported in a NAFLD rat model where severe inflammation was observed [32]. This might indicate the similarity of inflammation between IECs and hepatocytes. However, *PEMT* has been suggested to be predominantly expressed in hepatocytes. Thus, it is unknown whether the *PEMT* expression or the same change is plausible in intestinal cells. We failed to validate these predictions due to lack of samples for further analysis (qPCR).

Different patterns of fatty acid reactions were observed in all three groups of UC (see supplementary figure S6). Fatty acids play an important role in regulating cell-specific functions and immune responses [33].

Genes *ELOVL5* and *SCD* were predicted to change in the three groups while *FADS2* only changed in the UC refractory and treatment naïve group (see Table 3). Corresponding changes in gene expressions have been reported to be highly correlated with differential expressions of some immunity-related genes [34]. This might indicate immune activity in UC patients, even for the remission patients. *ELOVL3*, which is responsible for maintaining lipid homeostasis [35], was predicted to change in both refractory and treatment naïve group.

The lipid profiles of CD and UC in remission were similar to normal controls. This indicates resolution of the inflammation in these two groups and consequently a downregulated inflammasome. The inflammasome in IBD represents immune activation both in IECs and immune cells deeper in the intestinal mucosa representing an important first line of defence [36]. This in turn triggers a cascade of the main immune components: genome, transcriptome, proteome and metabolome including the lipidome. In a previous study, we found a significant increase of *IL17* gene activity in the mucosa of UC patients in remission compared to controls [9]. This could imply that a lipidomic, but not necessarily an immunological, remission is a prerequisite to obtain deep remission in IBD. However, this awaits further studies, but may have therapeutic implications.

One strength of the present study is the number of refractory patients combined with in depth analysis, state-of-the-art lipidomic methods. The comparisons between normal controls, remission, moderate to severe acute inflammation and a refractory state are unique and open for comprehensive evaluations. Furthermore, characterization of *TNF* gene expression provides a connection between lipid profiles and immunological response. However, it is not possible to infer any conclusions regarding non-response to vedoluzimab due to few observations (2 CD patients), although they seemed similar to the other refractory patients. They had both previously failed on antiTNFs. Given the exploratory nature of the study, a formal power analysis was not conducted.

The influence of diet on the lipid profile of the mucosa is uncertain. Phospholipids like sphingolipids and glycerophospholipids in the intestine are either formed de novo or are of dietary origin [14]. The data regarding diet were incomplete. Complete and more elaborate datasets on diet combined with fecal and serologic lipid levels could possibly be of value. Also, subgroup analysis of sampling location (small vs. large intestine) could have provided additional information, but was not feasible.However, regarding PC it has been proposed that the colon is largely dependent on de novo synthesis rather than absorption from bile and diet, in contrast to the small intestine [14]. Systemic steroids could also intererfere with the lipid profiles as some refractory IBD patients (5 UC and 2 CD) were taking these at inclusion.

As lipidomics are based on relative values and not absolute quantification of lipid species, a validation step in future studies would be to do targeted analyses with absolute quantification of the identified lipid metabolites in additional samples. This could possibly yield further insights into key molecules and events in treatment refractory IBD.

Of note, there were fewer significant differences in lipid profiles between UC refractory and UC remission than would be anticipated. A possible explanation could be ongoing healing processes in both states and altered immune mechanisms in a refractory mucosa.

A subdivision of treatment refractory IBD into primary and secondary non-responders could also be of value as the underlying cause of non-response could differ between these two groups. Unfortunately the present study was underpowered for subgroup analysis.

Finally, our study demonstrated particularly a relative heterogeneity in the clusters of data in CD refractory and treatment naïve groups (see supplementary Fig. 2). The transmural inflammation of CD with different sampling sites (small and large intestine) and varying cell types could be the cause of this. UC presents with a superficial inflammation, thus increasing the probability of sampling representative tissue. Also, different mechanisms between the two disease entities cannot be excluded due to lack of deep mucosal biopsies.

## Conclusion

The lipid profile of treatment refractory UC patients is different from those with newly diagnosed UC classified with moderate to severe active inflammation. This is of interest in the research field of IBD as it indicates dynamic processes of enduring inflammation and impaired mucosal barrier function. Furthermore, the mucosal lipid profile of IBD patients in remission is similar to the normal state. Taken together, these are novel findings which give new insights into the treatment refractory state of IBD and possible targets for treatment.

## Supplementary Information

Supplementary Material 1: Figure S1 Distribution of included patients. IBD: inflammatory bowel disease; UC: ulcerative colitis, CD: Crohn`s disease; PNR: primary non-response and; SNR: secondary non-response

Supplementary Material 2: Figure S2 Visualization of lipid profiles using UMAP

Supplementary Material 3: Figure S3 Volcano plot of change in lipids inUC refractory compared to normal controls andUC treatment naïve compared to normal controls. The estimate is calculated from generalized least squares coefficients and Benjamini-Hochberg procedure was used for multiple testing correction. Significance: non-significant; *p* < 0.05; *p* < 0.01. a Change of lipids: UC refractory. b Change of lipids: UC treatment naïve – normal

Supplementary Material 4: Figure S4 Lipid networks for UC remission, refractory and treatment naïve group compared to normal controls generated using BioPAN. Active lipids are represented as green nodes and active pathways are coloured with green shadow. Green and purple arrows indicate active and suppressed reactions with Z scores, respectively

Supplementary Material 5: Figure S5 Boxplot of PC:PE ratio in normal controls, CD and UC remission, refractory and treatment naïve groups. PC:PE ratio was calculated based on the lipids involved in the known reactive pathways. Specifically, the following lipids were included in the calculation of pathways and plot: PC: PC, PC, PC, PC, PC, PC, PC, PE: PE, PE, PE, PE, PE, PE, PE. A two-tailed *p* value below 0.05 was considered statistically significant

Supplementary Material 6: Figure S6 Fatty acid network for UC remission, refractory and treatment naïve group compared to normal controls generated using BioPAN. Active fatty acids are represented as green nodes and active pathways are coloured with green shadow. Green and purple arrows indicate active and suppressed reactions with Z scores, respectively

Supplementary Material 7: Figure S7 Volcano plot of change in lipids in a) CD refractory compared to normal controls, b) CD treatment naïve compared to normal controls and c) CD refractory compared to CD treatment naive. The estimate is calculated from generalized least squares coefficients and Benjamini-Hochberg procedure was used for multiple testing correction. Significance: non-significant; *p* < 0.05; *p* < 0.01

Supplementary Material 8: Figure S8 Lipid networks for CD refractory compared to normal controls generated using BioPAN. Active lipids are represented as green nodes and active pathways are coloured with green shadow. Green and purple arrows indicate active and suppressed reactions with Z scores, respectively

Supplementary Material 9.

Supplementary Material 10.

Supplementary Material 11.

Supplementary Material 12.

Supplementary Material 13.

Supplementary Material 14.

Supplementary Material 15.

## Data Availability

Data is provided within the manuscript or supplementary information files.

## Abbreviations

ASIB- study: Advanced Study of Inflammatory Bowel disease
CD: Crohn’s disease
CDAI: Crohn’s disease activity index
Cer: Ceramide
IBD: Inflammatory bowel disease
PC: Glycerophosphocholine
PE: Glycerophosphoethanolamine
PS: Glycerophosphoserines
SM: Sphingomyelin
TNF: Tumor necrosis factor
UC: Ulcerative colitis
UCDAI: Ulcerative colitis activity index
