# Supplementary figures and images for: Marked mucosal lipid shifts in treatment refractory inflammatory bowel disease: a lipidomic study
Source: BMC Gastroenterol. 2025 May 20;25:389. doi: 10.1186/s12876-025-03944-6 (PMC12093861; doi:10.1186/s12876-025-03944-6)

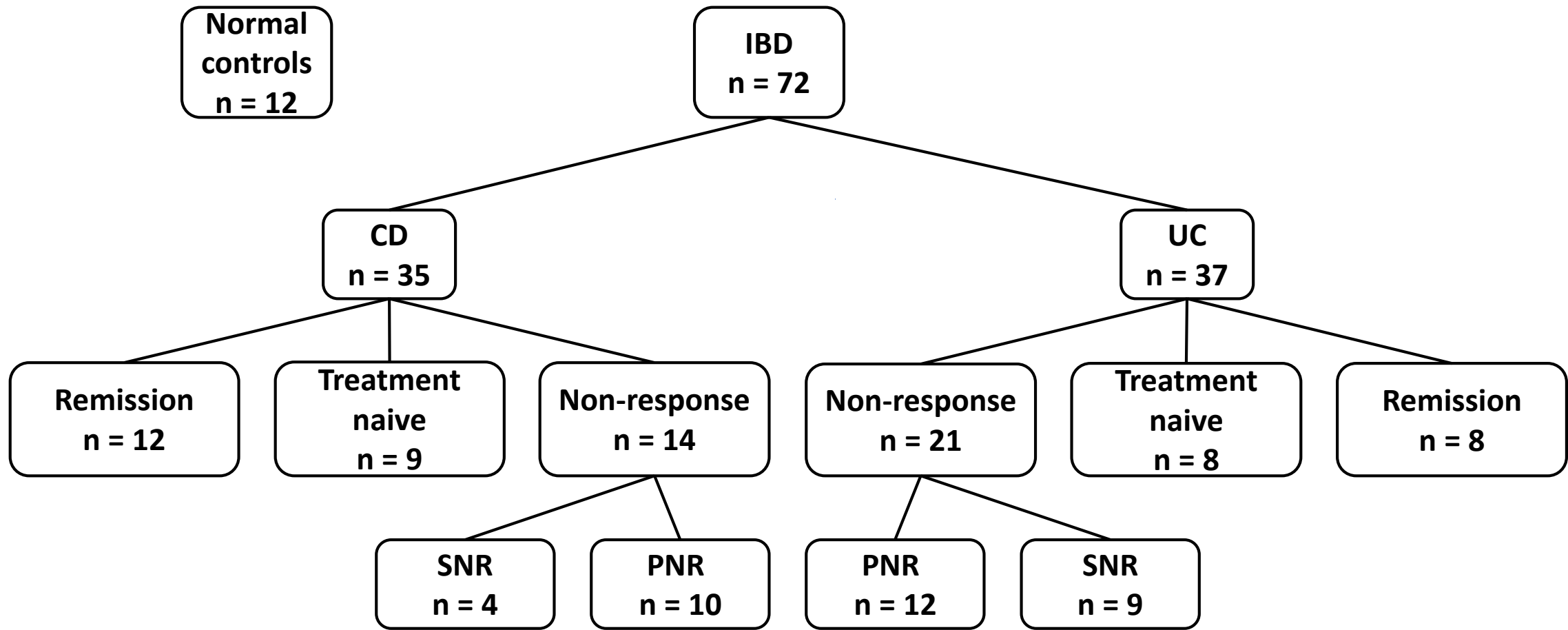

Supplement: Supplementary file 1 — Supplementary Material 1: Figure S1 Distribution of included patients. IBD: inflammatory bowel disease; UC: ulcerative colitis, CD: Crohn`s disease; PNR: primary non-response and; SNR: secondary non-response [file 12876_2025_3944_MOESM1_ESM.pdf]

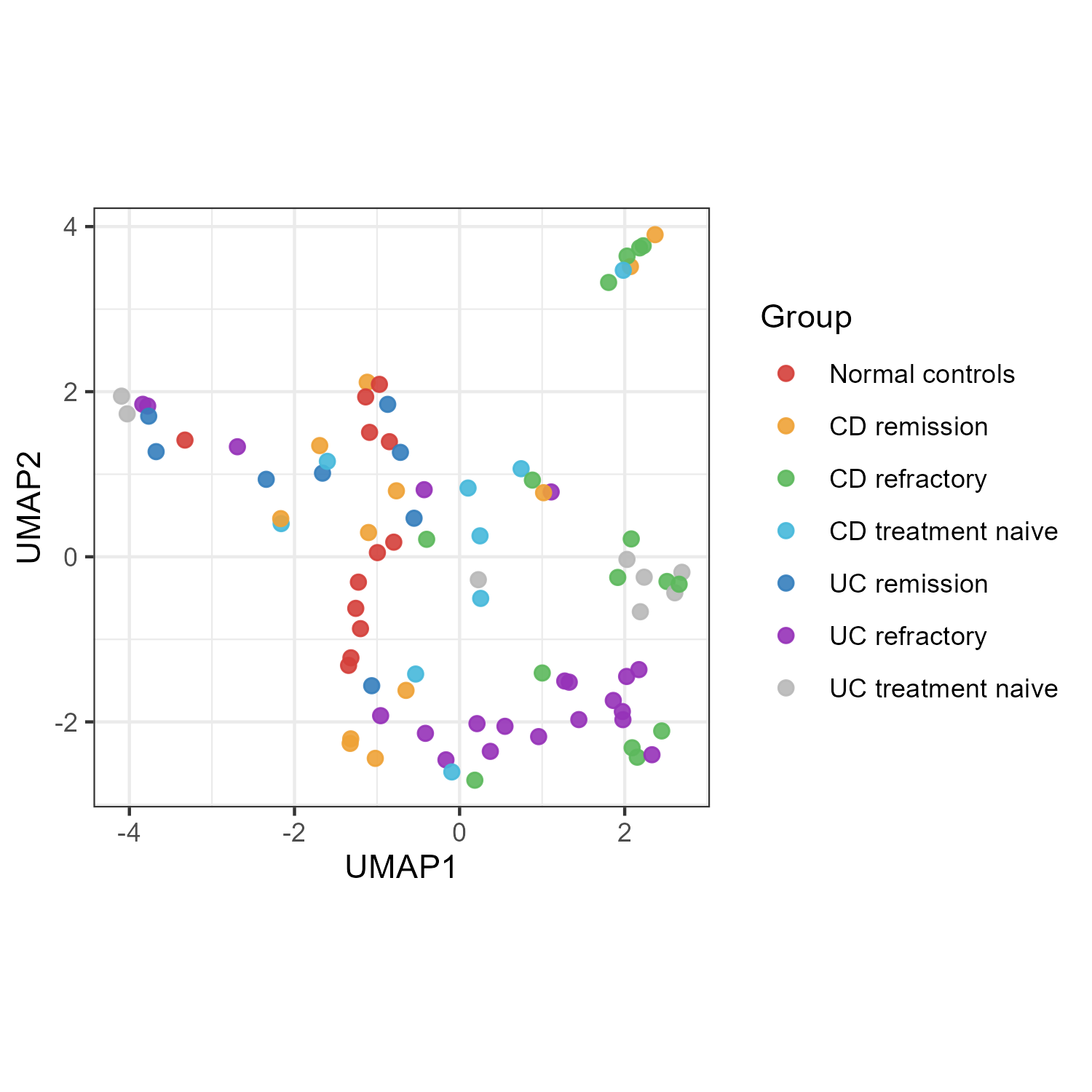

Supplement: Supplementary file 2 — Supplementary Material 2: Figure S2 Visualization of lipid profiles using UMAP [file 12876_2025_3944_MOESM2_ESM.png]

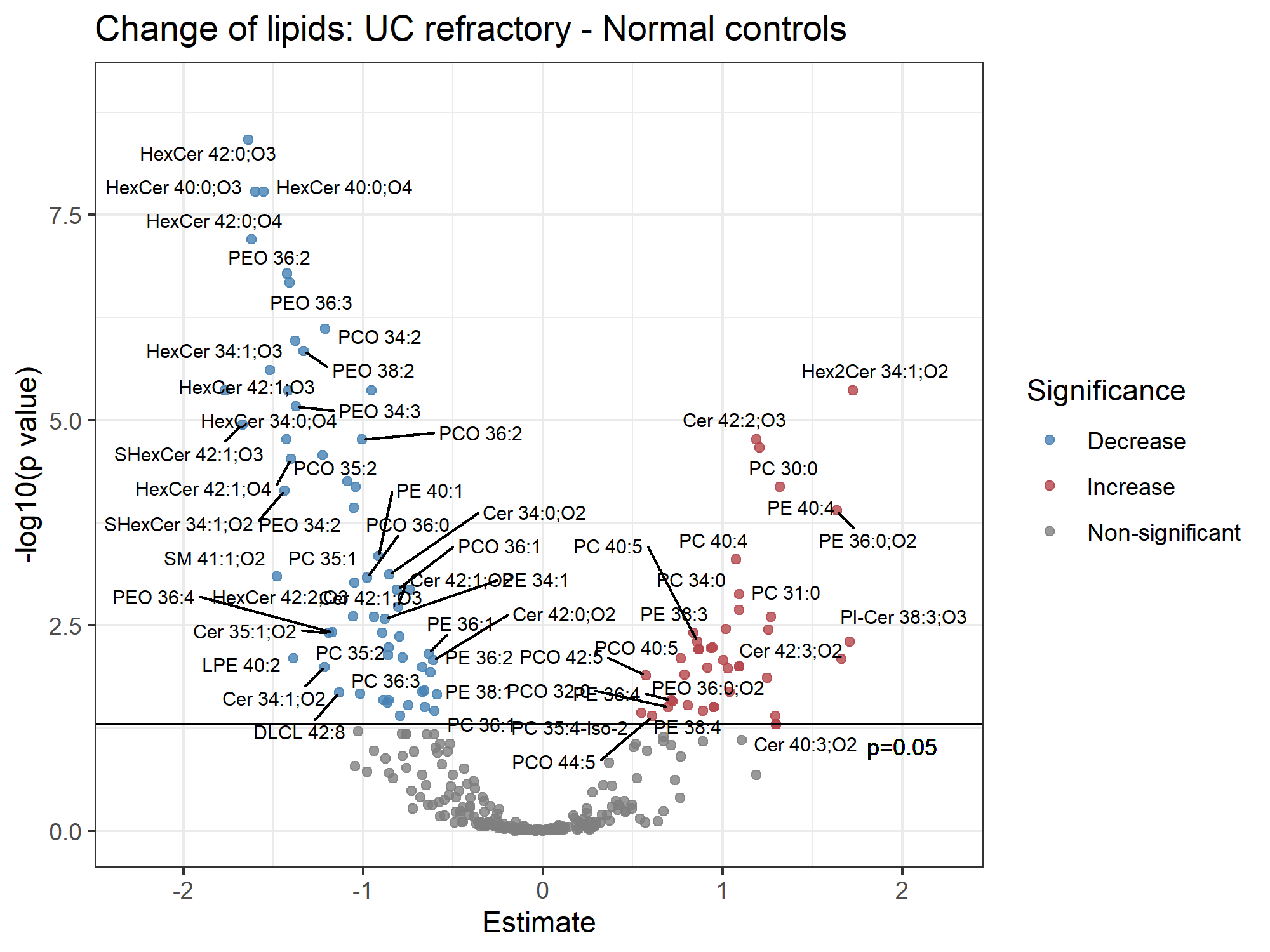

Supplement: Supplementary file 3 — Supplementary Material 3: Figure S3 Volcano plot of change in lipids inUC refractory compared to normal controls andUC treatment naïve compared to normal controls. The estimate is calculated from generalized least squares coefficients and Benjamini-Hochberg procedure was used for multiple testing correction. Significance: non-significant; p < 0.05; p < 0.01. a Change of lipids: UC refractory. b Change of lipids: UC treatment naïve – normal [file 12876_2025_3944_MOESM3_ESM.zip › Supplementary figure S3 a)_BMC.png]

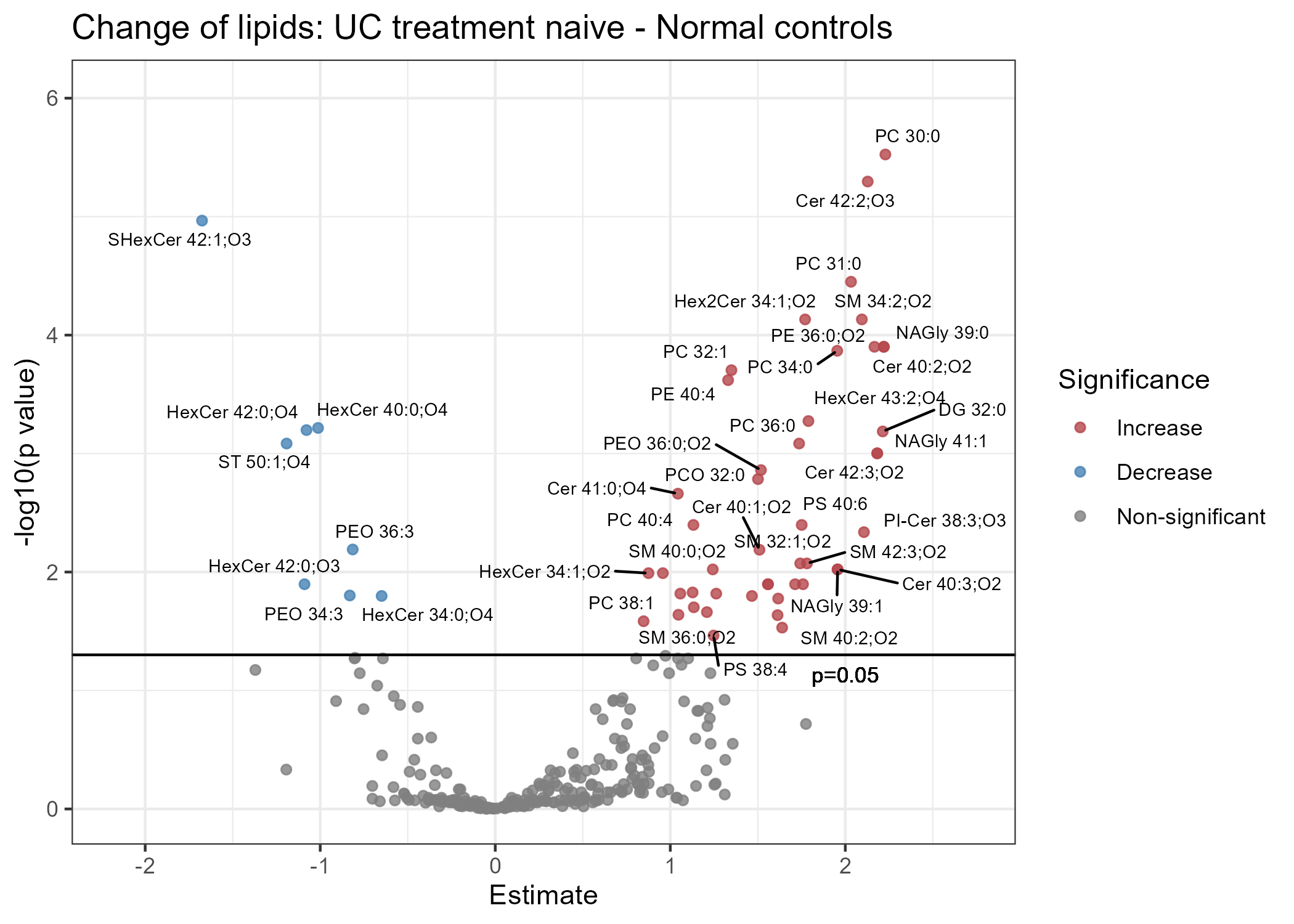

Supplement: Supplementary file 3 — Supplementary Material 3: Figure S3 Volcano plot of change in lipids inUC refractory compared to normal controls andUC treatment naïve compared to normal controls. The estimate is calculated from generalized least squares coefficients and Benjamini-Hochberg procedure was used for multiple testing correction. Significance: non-significant; p < 0.05; p < 0.01. a Change of lipids: UC refractory. b Change of lipids: UC treatment naïve – normal [file 12876_2025_3944_MOESM3_ESM.zip › Supplementary figure S3 b)_BMC.png]

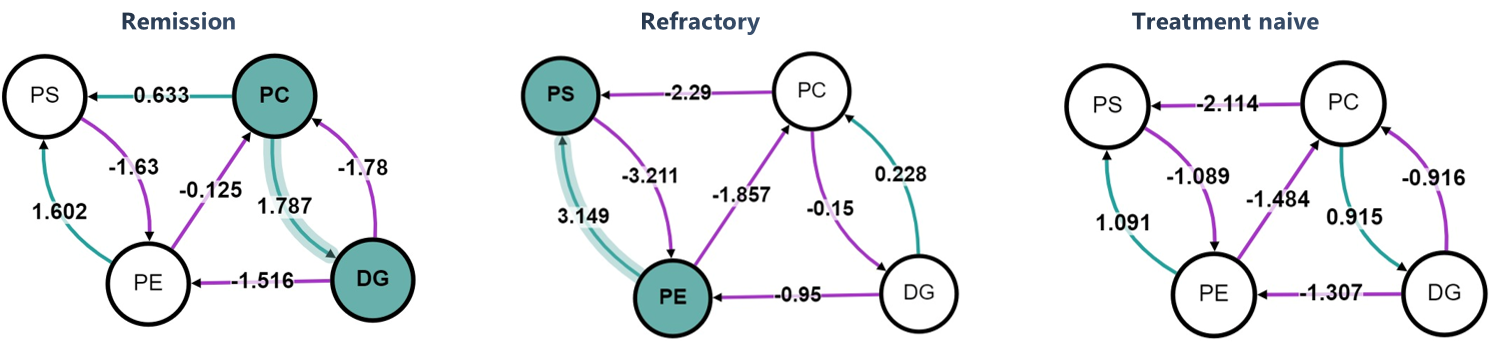

Supplement: Supplementary file 4 — Supplementary Material 4: Figure S4 Lipid networks for UC remission, refractory and treatment naïve group compared to normal controls generated using BioPAN. Active lipids are represented as green nodes and active pathways are coloured with green shadow. Green and purple arrows indicate active and suppressed reactions with Z scores, respectively [file 12876_2025_3944_MOESM4_ESM.png]

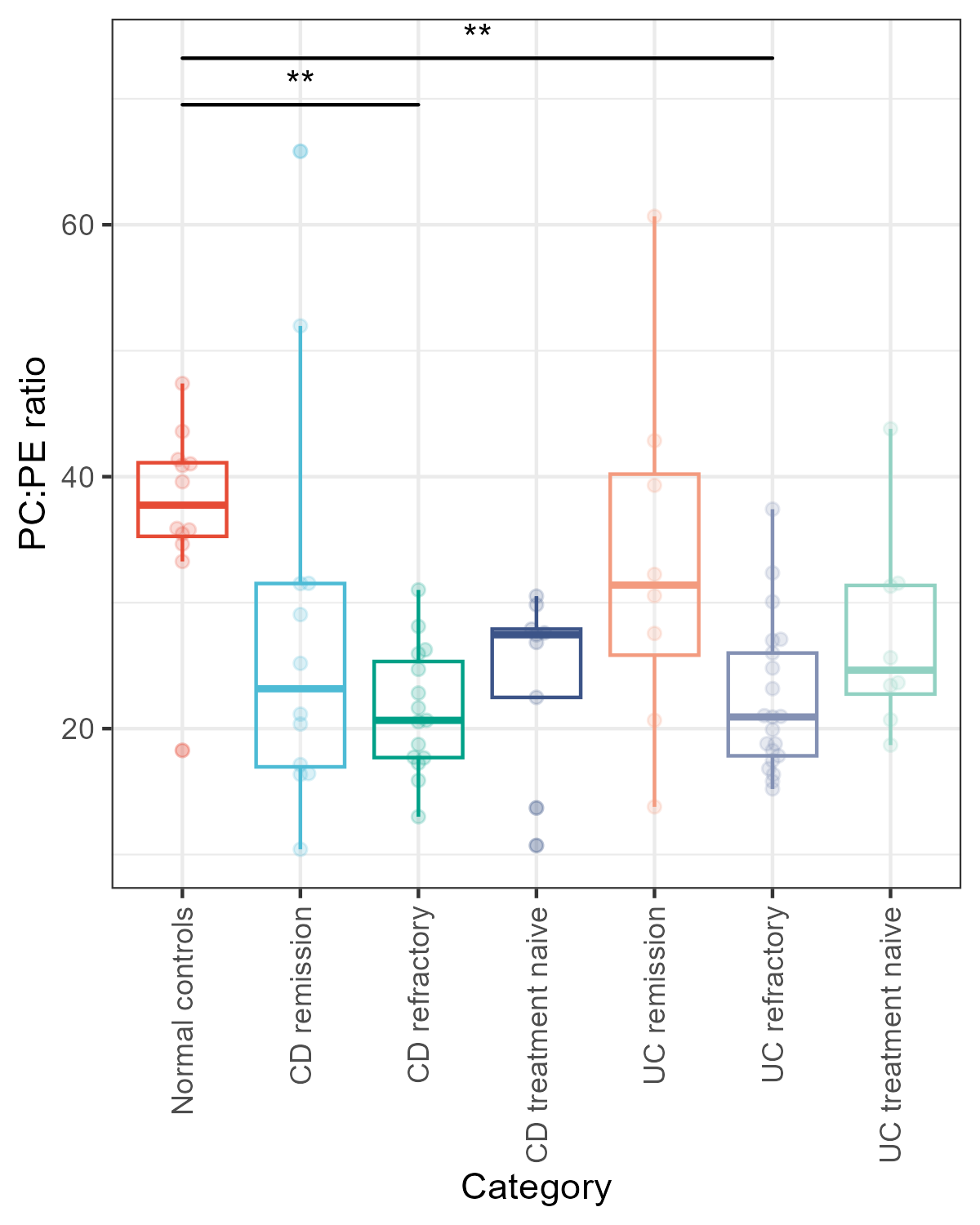

Supplement: Supplementary file 5 — Supplementary Material 5: Figure S5 Boxplot of PC:PE ratio in normal controls, CD and UC remission, refractory and treatment naïve groups. PC:PE ratio was calculated based on the lipids involved in the known reactive pathways. Specifically, the following lipids were included in the calculation of pathways and plot: PC: PC, PC, PC, PC, PC, PC, PC, PE: PE, PE, PE, PE, PE, PE, PE. A two-tailed p value below 0.05 was considered statistically significant [file 12876_2025_3944_MOESM5_ESM.png]

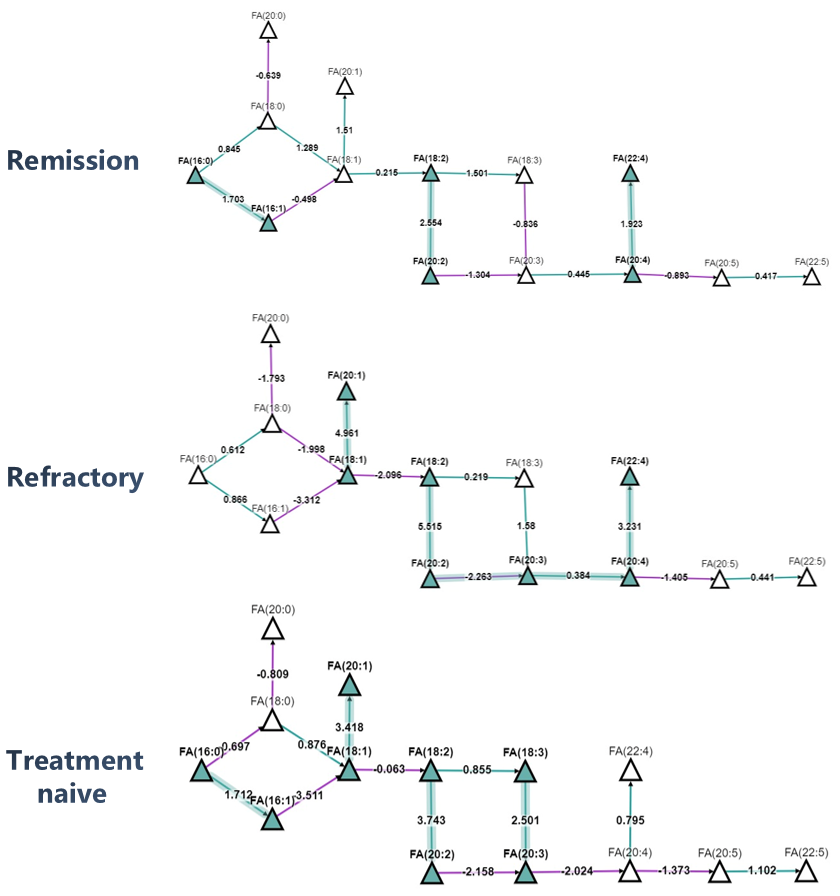

Supplement: Supplementary file 6 — Supplementary Material 6: Figure S6 Fatty acid network for UC remission, refractory and treatment naïve group compared to normal controls generated using BioPAN. Active fatty acids are represented as green nodes and active pathways are coloured with green shadow. Green and purple arrows indicate active and suppressed reactions with Z scores, respectively [file 12876_2025_3944_MOESM6_ESM.png]

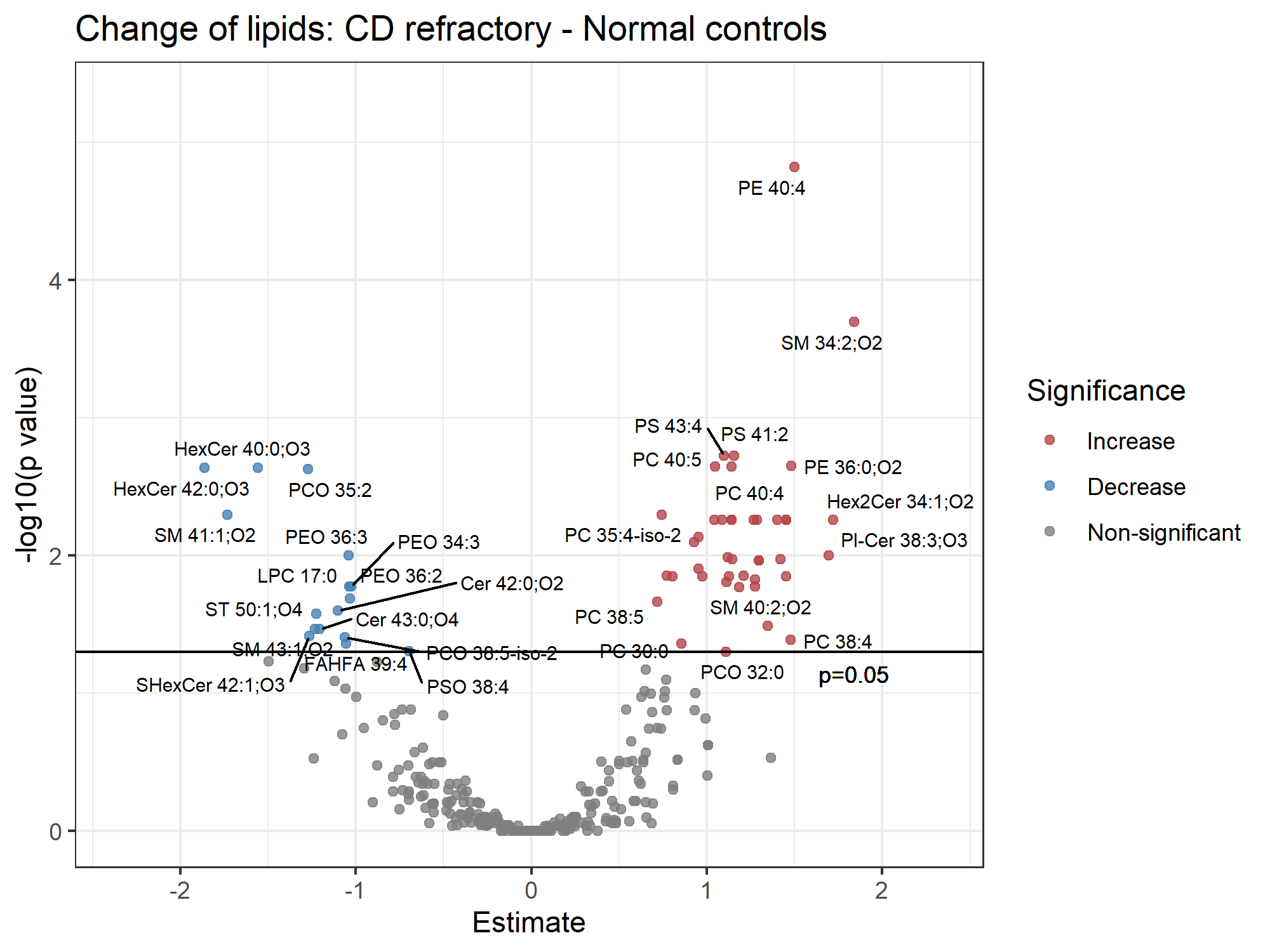

Supplement: Supplementary file 7 — Supplementary Material 7: Figure S7 Volcano plot of change in lipids in a) CD refractory compared to normal controls, b) CD treatment naïve compared to normal controls and c) CD refractory compared to CD treatment naive. The estimate is calculated from generalized least squares coefficients and Benjamini-Hochberg procedure was used for multiple testing correction. Significance: non-significant; p < 0.05; p < 0.01 [file 12876_2025_3944_MOESM7_ESM.zip › Supplementary figure S7 a)_BMC.png]

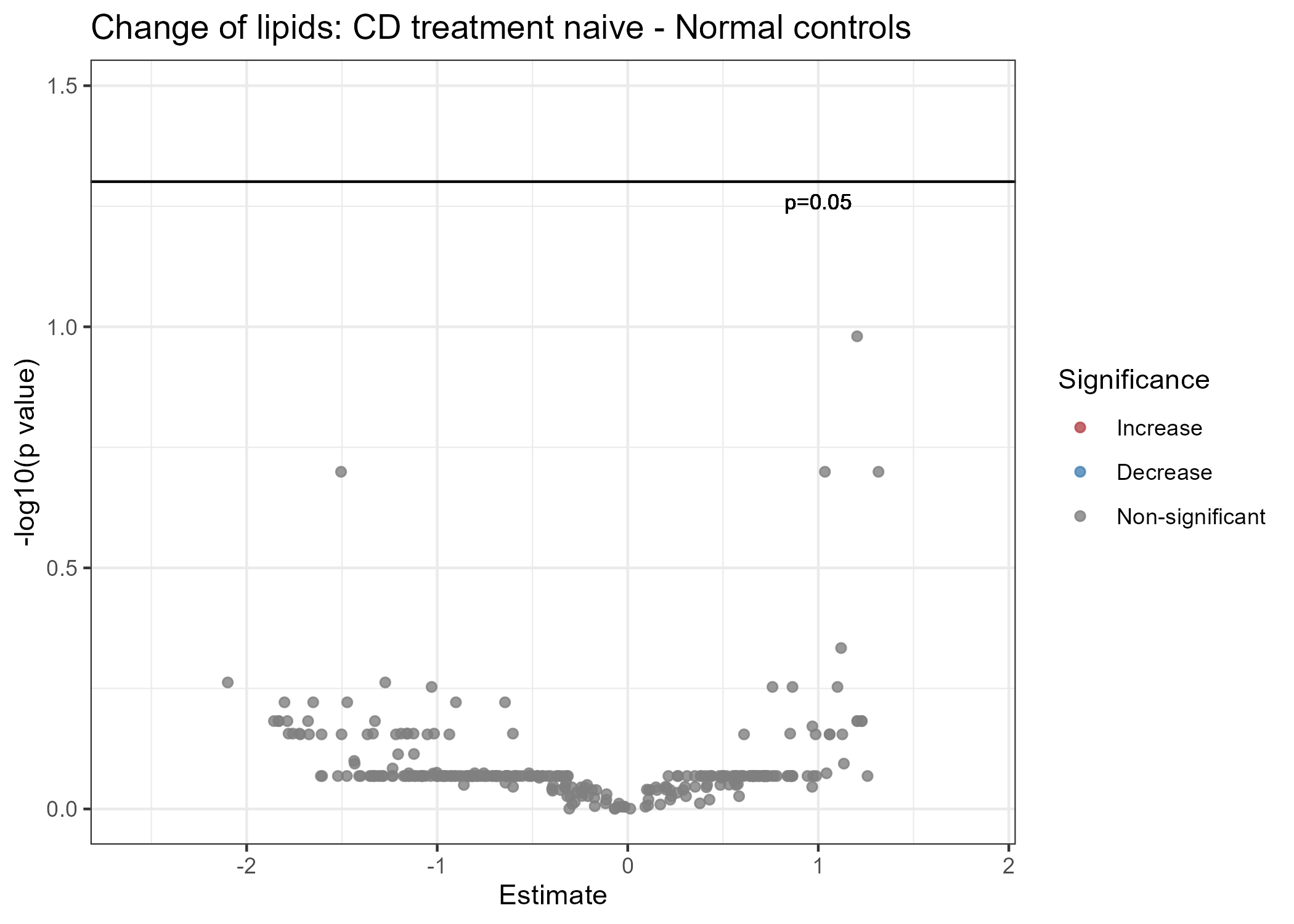

Supplement: Supplementary file 7 — Supplementary Material 7: Figure S7 Volcano plot of change in lipids in a) CD refractory compared to normal controls, b) CD treatment naïve compared to normal controls and c) CD refractory compared to CD treatment naive. The estimate is calculated from generalized least squares coefficients and Benjamini-Hochberg procedure was used for multiple testing correction. Significance: non-significant; p < 0.05; p < 0.01 [file 12876_2025_3944_MOESM7_ESM.zip › Supplementary figure S7 b)_BMC.png]

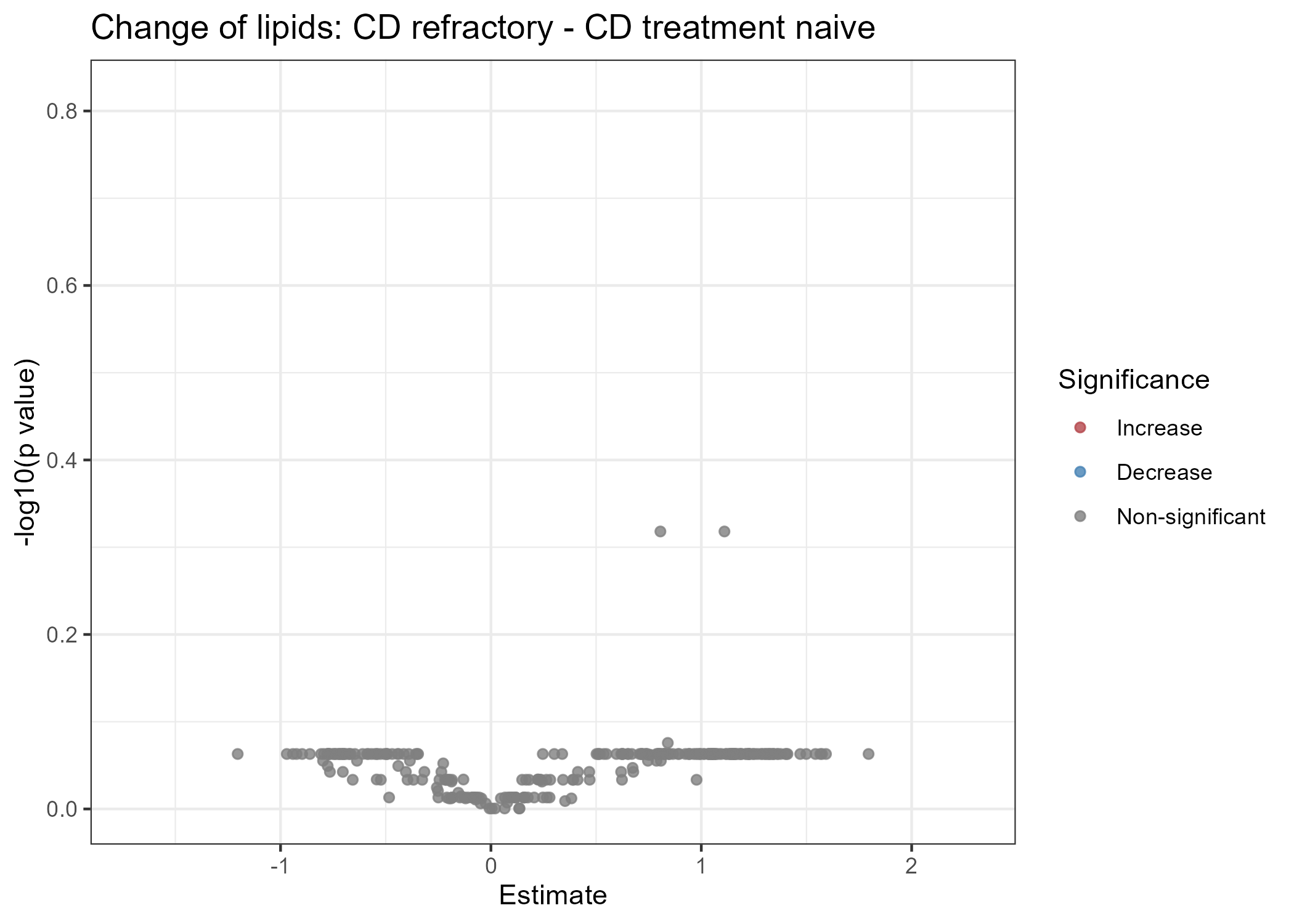

Supplement: Supplementary file 7 — Supplementary Material 7: Figure S7 Volcano plot of change in lipids in a) CD refractory compared to normal controls, b) CD treatment naïve compared to normal controls and c) CD refractory compared to CD treatment naive. The estimate is calculated from generalized least squares coefficients and Benjamini-Hochberg procedure was used for multiple testing correction. Significance: non-significant; p < 0.05; p < 0.01 [file 12876_2025_3944_MOESM7_ESM.zip › Supplementary figure S7 c)_BMC.png]

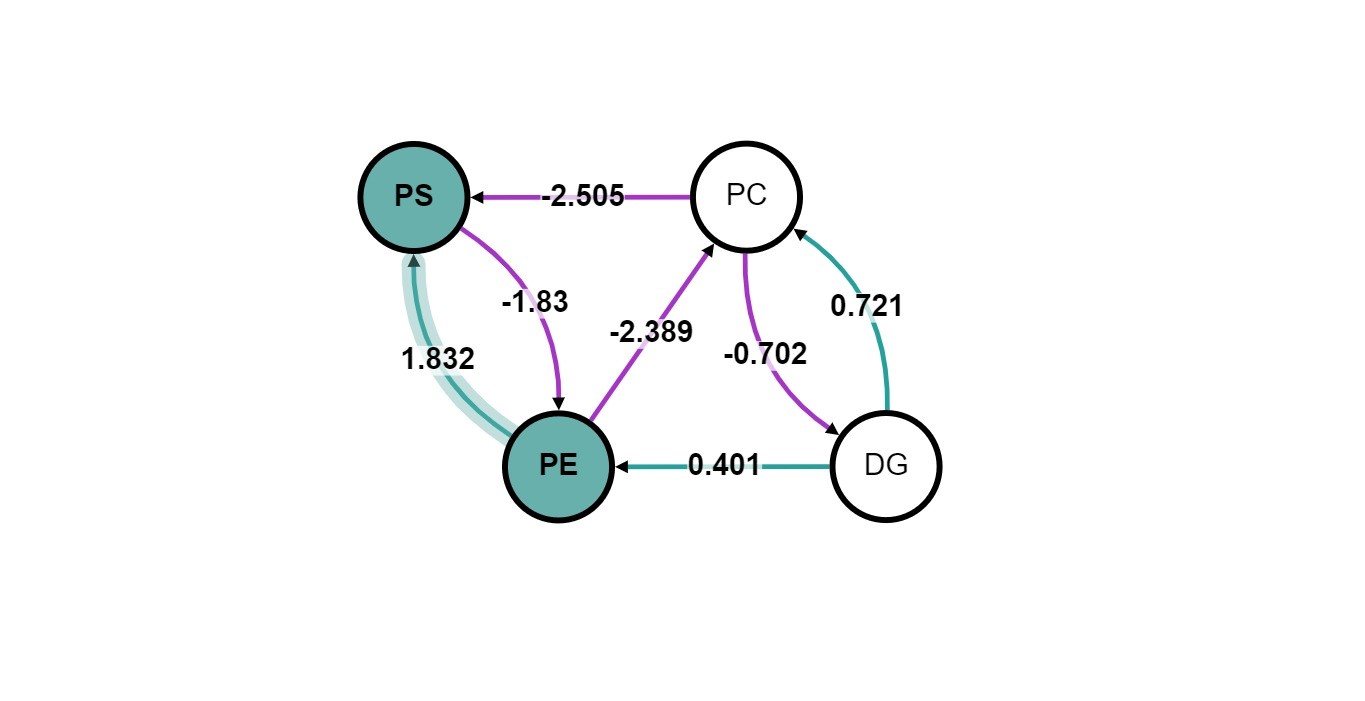

Supplement: Supplementary file 8 — Supplementary Material 8: Figure S8 Lipid networks for CD refractory compared to normal controls generated using BioPAN. Active lipids are represented as green nodes and active pathways are coloured with green shadow. Green and purple arrows indicate active and suppressed reactions with Z scores, respectively [file 12876_2025_3944_MOESM8_ESM.jpg]
